# Intraoperative Oliguria with Decreased SvO_2_ Predicts Acute Kidney Injury after Living Donor Liver Transplantation

**DOI:** 10.3390/jcm8010029

**Published:** 2018-12-28

**Authors:** Won Ho Kim, Hyung-Chul Lee, Leerang Lim, Ho-Geol Ryu, Chul-Woo Jung

**Affiliations:** 1 Department of Anesthesiology and Pain Medicine, Seoul National University Hospital, Seoul 03080, Korea; azong@hanmail.net (H.-C.L.); erange@snu.ac.kr (L.L.); hogeol@gmail.com (H.-G.R.); jungcwoo@gmail.com (C.-W.J.); 2 Department of Anesthesiology and Pain Medicine, Seoul National University College of Medicine, Seoul 03080, Korea

**Keywords:** acute kidney injury, urine output, hemodynamics, living-donor liver transplantation

## Abstract

Acute kidney injury (AKI) is a frequent complication after living donor liver transplantation (LDLT), and is associated with increased mortality. However, the association between intraoperative oliguria and the risk of AKI remains uncertain for LDLT. We sought to determine the association between intraoperative oliguria alone and oliguria coupled with hemodynamic derangement and the risk of AKI after LDLT. We evaluated the hemodynamic variables, including mean arterial pressure, cardiac index, and mixed venous oxygen saturation (SvO_2_). We reviewed 583 adult patients without baseline renal dysfunction and who did not receive hydroxyethyl starch during surgery. AKI was defined using the Kidney Disease Improving Global Outcomes criteria according to the serum creatinine criteria. Multivariable logistic regression analysis was performed with and without oliguria and oliguria coupled with a decrease in SvO_2_. The performance was compared with respect to the area under the receiver operating characteristic curve (AUC). Intraoperative oliguria <0.5 and <0.3 mL/kg/h were significantly associated with the risk of AKI; however, their performance in predicting AKI was poor. The AUC of single predictors increased significantly when oliguria was combined with decreased SvO_2_ (AUC 0.72; 95% confidence interval (CI) 0.68–0.75 vs. AUC of oliguria alone 0.61; 95% CI 0.56–0.61; *p* < 0.0001; vs. AUC of SvO_2_ alone 0.66; 95% CI 0.61–0.70; *p* < 0.0001). Addition of oliguria coupled with SvO_2_ reduction also increased the AUC of multivariable prediction (AUC 0.87; 95% CI 0.84–0.90 vs. AUC with oliguria 0.73; 95% CI 0.69–0.77; *p* < 0.0001; vs. AUC with neither oliguria nor SvO_2_ reduction 0.68; 95% CI 0.64–0.72; *p* < 0.0001). Intraoperative oliguria coupled with a decrease in SvO_2_ may suggest the risk of AKI after LDLT more reliably than oliguria alone or decrease in SvO_2_ alone. Intraoperative oliguria should be interpreted in conjunction with SvO_2_ to predict AKI in patients with normal preoperative renal function and who did not receive hydroxyethyl starch during surgery.

## 1. Introduction 

The incidence of acute kidney injury (AKI) after orthotopic liver transplantation has been reported to be as high as 64% [1,2,3,4]. AKI is an important complication associated with poor graft survival and increased mortality [1,2,5,6,7,8]. Furthermore, post-transplant AKI is associated with the development of chronic kidney disease [3,9,10].

Diagnosis of AKI is based on elevation of serum creatinine and/or decrease in urine output in the currently available KDIGO (Kidney Disease Improving Global Outcomes) criteria [7,11]. However, serum creatinine and urine output criteria have been criticized due to inaccurate reflection of glomerular filtration rate, insensitivity to detect acute change in renal function, [12,13] and being influenced by many factors including use of diuretics and volume status. In this regard, biomarkers to detect the development of AKI early after surgery have been investigated and showed promising performance for early detection [14]. However, the accuracy of the biomarkers are still controversial and there is still no biomarker with performance as good as that of troponin for detecting myocardial infarction [14].

The current urine output criteria of AKI suggest that a urine flow rate of <0.5 mL/kg/h lasting for more than 6 h indicates stage 1 AKI [7,11]. A rate of 0.3 mL/kg/h indicates stage 3 AKI. These criteria are widely regarded as the cutoff values to determine AKI in critically ill patients [15,16]. However, urine output criteria are regarded as unreliable in predicting AKI after surgery, because oliguria may develop during surgery due to decreased preload or nephrotoxic drug in addition to intrarenal oliguria [17]. AKI may develop in the absence of oliguria and oliguria may develop due to external obstruction of urinary tract [18,19]. Furthermore, during living donor liver transplantation (LDLT), urine output is influenced by many other factors, including baseline hepatorenal syndrome, cardiac preload condition, hemodynamics, sympathetic tone, and endocrine factors such as anti-diuretic hormones and aldosterone. Therefore, clinical significance of oliguria that is associated with postoperative AKI may be different from the currently available diagnostic criteria. 

The etiology of acute kidney injury during surgery includes hemodynamic instability that may result in decreased renal perfusion. During LDLT, intraoperative hemodynamic variables including continuous arterial pressure, cardiac index, and mixed venous oxygen saturation (SvO_2_) from pulmonary artery catheter are frequently monitored, although less invasive monitoring is increasingly used. Oliguria coupled with the deterioration in these hemodynamic variables may predict AKI better than oliguria alone because the other causes of oliguria that are not associated with AKI could be excluded. Oliguria developed from poor renal perfusion and oxygen delivery may be more strongly associated with AKI after LDLT. 

Therefore, in the present study, we aimed to investigate the specific impact of intraoperative oliguria on the risk of AKI after LDLT determined by KDIGO criteria. We also hypothesized that oliguria coupled with low mean arterial blood pressure, low cardiac index, or low SvO_2_ may predict AKI after LDLT better than oliguria alone. The objective of this retrospective study was to compare the association of intraoperative oliguria alone with oliguria observed with hemodynamic deterioration on the risk of AKI after LDLT. 

## 2. Materials and Methods

### 2.1. Study Design

This retrospective observational study was approved by the institutional review board of Seoul National University Hospital (1608-073-784). We reviewed the electronic medical records of 1211 consecutive adult patients who had undergone liver transplantation at our institution between 2004 and 2015. The need for informed consent was waived because the study had a retrospective design. Patients who received deceased donor liver transplantation (*n* = 367), those who had baseline renal dysfunction with an estimated glomerular filtration rate of <60 mL/min/1.73m^2^ and/or with hepatorenal syndrome (*n* = 73), and those who received hydroxyethyl starch during surgery (*n* = 188) were excluded from the analysis. The remaining 583 patients who had undergone LDLT were analyzed. 

### 2.2. Anesthesia, Surgical Technique and Immunosuppression

During the study period, the anesthesia protocol of our institution was as follows. Anesthesia was induced and maintained using propofol, rocuronium, and sevoflurane. Volume-controlled ventilation was maintained, with a tidal volume of 6–8 mL kg^−1^ and a FiO_2_ of 0.5. Arterial-line catheters were inserted into the radial and femoral arteries. A Swan-Ganz catheter was inserted through a 9-Fr Advanced Venous Access catheter (Edward Lifesciences, Irvine, CA, USA) that was placed in the right internal jugular vein. Continuous cardiac index and right ventricle-associated variables were monitored using the Vigilance II monitor (Edward Lifesciences, Irvine, CA, USA). The cause of hypotension was determined on the basis of cardiac index, stroke volume variation, SvO_2_ and systemic vascular resistance and was treated using either (1) ephedrine and continuous dopamine, (2) phenylephrine and norepinephrine, and/or (3) epinephrine. Donor grafts were prepared using a histidine-tryptophan-ketoglutarate solution. The piggyback technique was used to anastomose the graft and donor vessels. End-to-end anastomosis of the hepatic artery and duct-to-duct anastomosis of the bile duct were carried out in succession. During surgery, immunosuppression was induced using 500 mg of methylprednisolone (Solumedrol, Pfizer, Ballerup, Denmark) and 20 mg of intravenous basiliximab (Simulect, Novartis Pharma B.V., Arnhem, The Netherlands). During the postoperative period, immunosuppression was induced using calcineurin inhibitors of either tacrolimus or cyclosporine with mycophenolate mofetil. 

When intraoperative oliguria occurred during the study period, we administered crystalloid solutions to increase preload when surgical bleeding is evident or preload indices including central venous pressure, pulmonary artery occlusion pressure, and/or right ventricular end-diastolic volume indicated intravascular hypovolemia. Vasopressor was used to increase renal perfusion pressure when urine output decreased with euvolemia. Diuretics were used only when intravascular volume overload was suspected with elevated central venous pressure, pulmonary artery occlusion pressure, and/or right ventricular end-diastolic volume. Diuretics were not used to treat intraoperative oliguria. 

The following crystalloid fluids were used to optimize the preload during LDLT before 2010: lactated Ringer’s solution, normal saline, and plasmalyte, and hydroxyethyl starch (Voluven, Fresenius Kabi, Germany). The administration rate of crystalloid during LDLT was adjusted according to the hemodynamic preload index of central venous pressure, pulmonary artery occlusion pressure, and stroke volume variation. The hematocrit threshold for red blood cell transfusion was 20% during LDLT throughout the study period. Intraoperative diuretics (furosemide; 10–20 mg) were used in patients with persistent positive fluid balance and when volume overload was suspected due to a pulmonary artery wedge pressure greater 18 mmHg. 

### 2.3. Data Collection

Based on previous literature, demographic and perioperative variables known to be related to postoperative renal dysfunction or AKI were collected [1,4,5,20,21,22]. Preoperatively, the Model for End-stage Liver Disease (MELD) score, the Child-Turcotte-Pugh (CTP) score, and the Child classification were determined for all recipients [23]. The following variables were also collected: history of hypertension, diabetes mellitus, ABO blood type incompatibility, preoperative serum albumin, preoperative diuretic medication, graft macrosteatosis, graft ischemic time, postoperative platelet count, intraoperative blood loss, intraoperative transfusion volume, intraoperative diuretics use, and mean tacrolimus trough concentration during the first week after surgery [20,24]. The mean urine output during surgery was calculated by averaging the intraoperative data obtained from the electronic medical records. Urine output was measured hourly in a urine bag connected to a Foley catheter. 

The resting arterial blood pressure before anesthesia induction was used as baseline blood pressure. The initially measured cardiac index and SvO_2_ by pulmonary artery catheter and the Vigilance II monitor were used as a baseline. Sudden decrease in mean arterial pressure, cardiac index, and SvO_2_ lower than 20% from baseline for at least one measurement was identified and recorded as a categorized variable. 

The primary outcome variable was postoperative AKI, as defined using the KDIGO criteria, which have been validated in patients undergoing LDLT [10]. We determined postoperative AKI based on the maximal change in serum creatinine level during the first seven postoperative days [25]. No urine output criteria were used due to the purpose of our study. Although the oliguria criteria of KDIGO requires that oliguria last more than 6 h, oliguria was determined based on the average urine out during surgery, regardless of its duration, in our study. 

### 2.4. Statistical Analysis 

SPSS software version 23.0 (IBM Corp., Armonk, NY, USA) and STATA/MP version 15.1 (StataCorp, College Station, TX, USA) were used to analyze the data. In all analyses, *p*-values < 0.05 were considered statistically significant. The Kolmogorov-Smirnov test was used to determine the normality of the data. All the continuous covariates listed in Table 1 followed non-parametric distribution. To compare all continuous variables in Table 1 and the urine output between those with and without AKI, the Mann-Whitney U test was used. Data were missing in fewer than 5% of records. We imputed the missing values according to the incidence of missing data. If the incidence of missing was < 1%, the missing data were substituted by the mean for continuous variables and by the mode for incidence variables. Missing values of variables with a ratio of missing data >1% and <5% were replaced by hot-deck imputation. 

The following is a summary of our statistical analysis. First, we determined the cutoff of intraoperative mean urine output by multivariable logistic regression analysis. Second, according to oliguria using the cutoff, we compared the predictive ability of the oliguria alone with that of oliguria coupled with a decrease in mean arterial pressure, cardiac index, and SvO_2_. Third, we evaluated whether the oliguria coupled with hemodynamic derangement could enhance the AUC of the multivariable prediction model for AKI after LDLT. 

Multivariable logistic regression analysis was performed (1) to find an optimal cutoff of oliguria, and (2) to evaluate the association between perioperative variables and postoperative AKI. The following variables were considered potential risk factors of AKI after LDLT in addition to oliguria and hemodynamic variables: age, sex, body-mass index, year of operation, MELD score, CTP score, hypertension, diabetes mellitus, preoperative hemoglobin level, preoperative serum albumin, preoperative serum creatinine, ABO blood type incompatibility, operation time, graft ischemic time, preoperative diuretics use, intraoperative blood loss, intraoperative crystalloid and colloid administration, transfusion amount and total dose of diuretics administered during surgery. We did not perform any univariable screening before the multivariable analysis. A *p*-value < 0.20 was used to select for significant predictors in the multivariable analysis, with the backward stepwise variable selection. We also performed multivariable logistic regression analysis without stepwise variable selection. 

Three different cutoffs of intraoperative mean intraoperative urine flow rate including 1.0, 0.5, and 0.3 mL/kg/h were evaluated, because oliguria less than these cutoffs was significantly associated with postoperative AKI in our preliminary multivariable logistic regression analysis using perioperative baseline variables with mean urine output with different cutoffs (Supplemental Table S1).

To compare the predictive ability of oliguria using these cutoffs with that of oliguria coupled with three hemodynamic variables, the area under the receiver operating characteristic curve (AUC) was calculated for every single potential predictor. Also, performance measurements including sensitivity, specificity, positive predictive value (PPV), and negative predictive value (NPV) were compared. 

Multivariable logistic regression analysis was performed again by adding the predictors of oliguria coupled with hemodynamic variables. Then, the AUCs of a multivariable prediction model with and without these oliguria coupled with hemodynamic variables were compared to evaluate whether these combined predictors could increase the predictive ability of multivariable prediction model. The comparison between AUCs was performed using De Long’ s method [26]. 

## 3. Results

During the first postoperative week, AKI, as determined using the KDIGO criteria, occurred in 205 patients (35.2%), and stage ≥2 AKI occurred in 43 patients (7.4%). Patient demographics and perioperative variables were compared between the patients with and without AKI in Table 1. The distribution of mean intraoperative mean urine flow rate is shown in Supplemental Figure S1. The median (interquartile ranges) of mean urine output were 1.15 (0.76–1.71) for those with AKI and 1.36 (0.89–2.08) for those without AKI. The mean intraoperative urine output was significantly lower in patients with AKI than in those without AKI (*p* = 0.007).

The odds ratios, 95% CI and their *p*-values according to oliguria with different cutoffs are shown in Supplemental Table S1. Multivariable logistic regression analysis showed that oliguria <0.5 and <0.3 mL/kg/h were significantly associated with AKI. 

The performances to predict postoperative AKI of a single variable of oliguria, a single variable of 20% decrease in hemodynamic variables, and their combined variables were compared in terms of AUC, sensitivity, specificity, PPV, and NPV (Table 2). Three different cutoffs of 0.3, 0.5, and 1.0 mL/kg/h were used, and hemodynamic variables included mean arterial pressure, cardiac index, and SvO_2_. AUC was largest for oliguria <0.5 mL/kg/h with SvO_2_ reduction (AUC = 0.72, 95% CI 0.68–0.75). 

**Figure 1:**
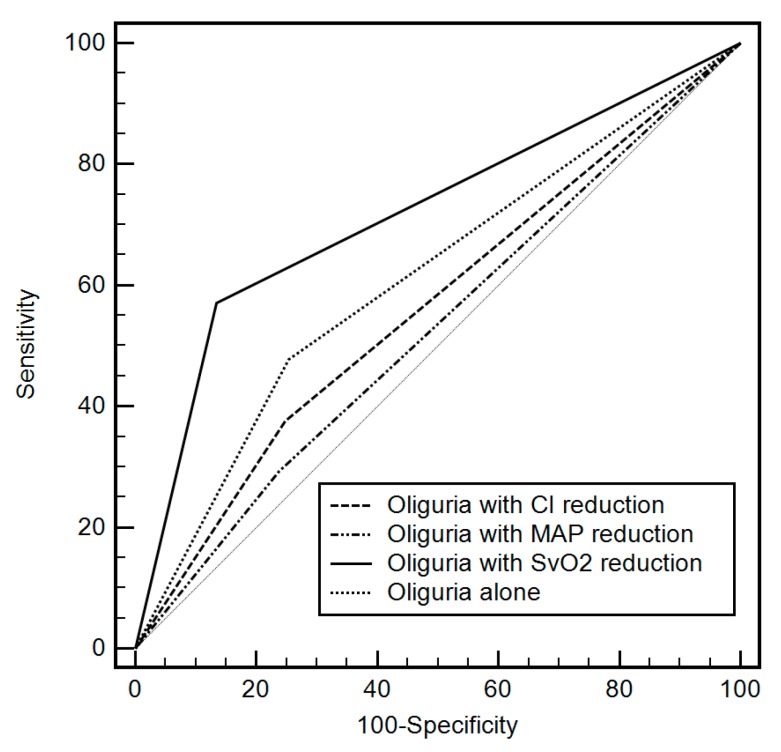
Comparison of area under the receiver operating characteristic curve between the four predictors of oliguria alone and oliguria coupled with hemodynamic variables including mean arterial pressure (MAP), cardiac index (CI), and mixed venous oxygen saturation (SvO_2_). Oliguria used a cutoff of 0.5 mL/kg/h and hemodynamic variables used 20% reduction from their baseline values. AUC of oliguria ≤ 0.5 mL/kg/h with SvO_2_ 20% reduction was significantly greater than all other variables (oliguria coupled with SvO_2_ reduction: AUC 0.72, 95% CI 0.68–0.75 vs. oliguria alone: 0.60, 95% CI 0.56–0.64, *p* < 0.001; vs. oliguria coupled with mean arterial pressure reduction: 0.53, 95% CI 0.49–0.57, *p* < 0.001; vs. oliguria coupled with cardiac index reduction: 0.56, 95% CI 0.52–0.60, *p* < 0.001).

The AUCs of four single predictors of oliguria <0.5 mL/kg/h alone, oliguria coupled with mean arterial pressure reduction, oliguria with cardiac index reduction, and oliguria with SvO_2_ reduction were compared in Figure 1. AUC of oliguria ≤0.5 mL/kg/h with SvO_2_ 20% reduction was significantly greater than all other variables (oliguria with SvO_2_ reduction: AUC 0.72, 95% CI 0.68–0.75 vs. oliguria alone: 0.60, 95% CI 0.56–0.64, *p* < 0.001; vs. oliguria with mean arterial pressure reduction: 0.53, 95% CI 0.49–0.57, *p* < 0.001; vs. oliguria with cardiac index reduction: 0.56, 95% CI 0.52–0.60, *p* < 0.001). 

The results of multivariable logistic regression analysis to predict postoperative AKI with and without including oliguria <0.5 mL/kg/h coupled with SvO_2_ reduction are shown in Table 3. The Nagelkerke’s *R*^2^ of the model with and without oliguria with SvO_2_ reduction were 0.16 and 0.39, respectively. Both models showed good calibration (Hosmer-Lemeshow goodness of fit: chi-square = 2.33, 8.76 and *p* = 0.264, 0.567, respectively). The results of multivariable logistic regression analysis without stepwise variable selection are shown in Supplemental Table S1.

**Figure 2:**
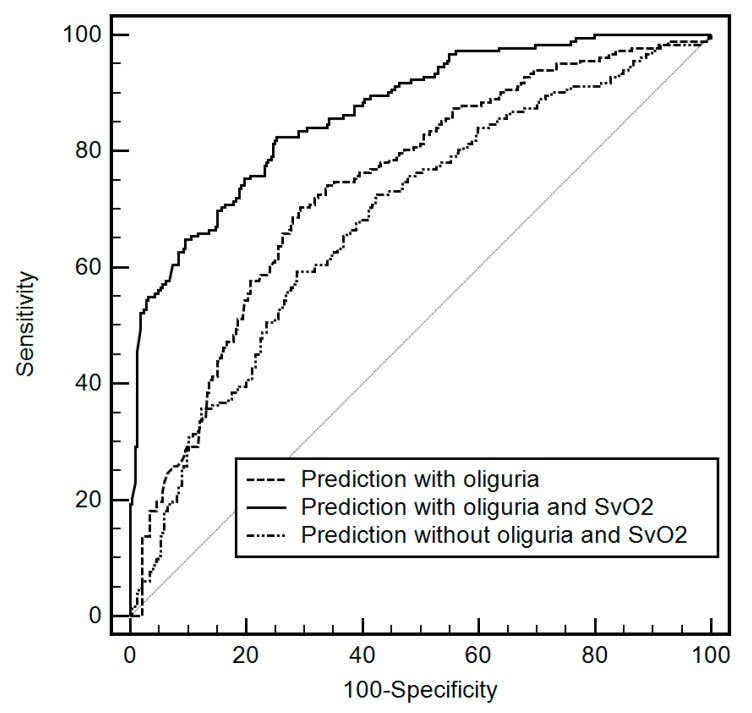
Comparison of area under the receiver operating characteristic curve between the multivariable prediction models (1) without oliguria or mixed venous oxygen saturation (SvO_2_) reduction, (2) with oliguria, and (3) with oliguria coupled with SvO_2_ reduction. AUC of model with oliguria coupled with SvO_2_ reduction was significantly greater than AUCs of model without SvO_2_ reduction (AUC with oliguria coupled with SvO_2_ reduction: 0.87, 95% CI 0.84–0.90 vs. AUC without oliguria or SvO_2_ reduction: 0.68, 95% CI 0.64–0.72, *p* < 0.0001; vs. AUC with oliguria: 0.73, 95% CI 0.69–0.77, *p* < 0.0001).

The AUCs of (1) the multivariable prediction without oliguria or SvO_2_, (2) multivariable prediction with oliguria <0.5 mL/kg/h, and (3) multivariable prediction with oliguria <0.5 mL/kg/h coupled with SvO_2_ reduction are compared in Figure 2 (AUC of oliguria coupled with SvO_2_ reduction: 0.87, 95% CI 0.84–0.90 vs. AUC without oliguria or SvO_2_ reduction: 0.68, 95% CI 0.64–0.72, *p* < 0.0001; vs. AUC with oliguria: 0.73, 95% CI 0.69–0.77, *p* < 0.0001). 

## 4. Discussion

To our knowledge, the present study was the first to evaluate the association between intraoperative mean urine output, hemodynamic variables, and AKI after LDLT. Although there was a significant association between oliguria <1.0, <0.5 and <0.3 mL/kg/h and risk of AKI, the predictive ability of oliguria alone was relatively poor and did not add substantial additional discriminative power in multivariable prediction. However, when oliguria developed in association with decreased SvO_2_, the AUC of a single predictor significantly increased, while oliguria coupled with mean arterial blood pressure or cardiac index failed to increase the AUC. These results suggested that oliguria accompanied by a reduction in SvO_2_ during LDLT may be more strongly associated with the development of AKI than oliguria or reduction in SvO_2_ alone. The AUC of multivariable prediction showed similar results and the model including oliguria coupled with decreased SvO_2_ showed significantly higher performance in terms of AUC and Nagelkerke’s *R*^2^. Although oliguria alone is not specific and has little value in predicting AKI after LDLT, oliguria coupled with a decrease in cardiac output or SvO_2_ could significantly increase the sensitivity and predictive ability for AKI after LDLT. However, our results should not be applied to the patients with poor baseline renal function including hepatorenal syndrome and the patients who received hydroxyethyl starch during surgery. Also, our results should be interpreted cautiously, because our oliguria was determined by the intraoperative mean value and the duration of oliguria was not considered. 

The urine output criteria of the RIFLE criteria (Risk, Injury, Failure, Loss of function, and End-stage renal disease) and the AKIN (AKI network) criteria are widely used to diagnose AKI [7]. The clinical implications of the oliguria criteria have been validated in a critical care setting [27,28]. However, intraoperative oliguria is regarded as less predictive of postoperative AKI compared to non-surgical settings [29]. AKI determined by serum creatinine may develop without oliguria, and AKI may not develop despite the intraoperative oliguria. Furthermore, recent studies have suggested that the urine output criteria for AKI in surgical patients may be different from the previous commonly used value of 0.5 mL/kg/h [30,31]. 

Several studies have attempted to derive urine output thresholds that identify AKI in surgical patients [30,31,32]. Using a methodology that was similar to ours, one retrospective study in cardiac surgical patients undergoing cardiopulmonary bypass identified a urine flow rate of <1.5 mL/kg/h as a cutoff that was associated with AKI risk [30]. Another retrospective study involving major abdominal surgery reported that <0.3 mL/kg/h was the threshold of the risk of AKI [31]. As such, the threshold urine output to diagnose AKI may vary depending on the surgical setting. Therefore, we first identified the optimal cutoff of oliguria that is significantly associated with AKI after LDLT in our study sample. However, in these studies, the predictive ability of intraoperative oliguria has not been evaluated fully. 

Along with the usual <0.5 or <0.3 mL/kg/h cutoff of oliguria, our analysis suggested significant associations between low urine flow rate of <1.0 mL/kg/h and risk of AKI after LDLT. We evaluated the cutoff of oliguria up to 3.0 mL/kg/h because previous studies reported different cutoffs of oliguria during surgery [30,31,32] (Supplemental Table S2). The significant threshold of <1.0 mL/kg/h may be influenced by the intraoperative diuretics use. More than 20% of our patients received diuretics before or intraoperatively. Our subgroup analysis after excluding all patients who administered diuretics pre- or intraoperatively (*n* = 435) showed that <1.0 mL/kg/h was not significantly associated with postoperative AKI (Supplemental Table S3). 

The performance of oliguria in predicting AKI defined by creatinine criteria was poor at any cutoff (Table 2). AUC was only between 0.55 to 0.60, with low sensitivity and specificity. Addition of oliguria <1.5 mL/kg/h did not significantly improve the predictive ability of multivariable prediction model for AKI. It seems that oliguria during LDLT is neither a sensitive nor specific marker of AKI—i.e., the patients with the same mean urine flow rate may or may not develop AKI. Although hemodynamic variables of SvO_2_ showed a slightly better performance as a single predictor, the other hemodynamic variables did not. However, oliguria coupled with a decrease in SvO_2_ showed a significantly better performance as a single predictor and enhanced the predictive ability of multivariable prediction when included in the multivariable logistic model.

A low SvO_2_ may suggest poor oxygen delivery to the kidney [33]. During LDLT, decreased preload by intraoperative bleeding may decrease cardiac output and thereby impair renal perfusion. An ischemia/reperfusion injury of the kidney may develop especially during liver graft reperfusion [34]. A low SvO_2_ during LDLT reflects decreased oxygen delivery by low cardiac output to the major organs including kidney. When oliguria developed in a patient who experienced a decrease in SvO_2_, the oliguria of the patient is more likely to be caused by poor renal perfusion and oxygen delivery to the kidney. Meanwhile, the oliguria alone may not be associated with poor oxygen delivery to the kidney but associated with an extrarenal cause such as a transient decrease in preload or urinary tract obstruction.

However, decrease in cardiac index or mean arterial pressure combined with oliguria did not increase AUC to predict AKI after LDLT. Mean arterial pressure is not a sensitive marker to measure the cardiac output and oxygen delivery to the major organ especially in patients with significant cirrhosis or during and after reperfusion with unequal vasodilation [35]. Cardiac index could be more important than mean arterial pressure. However, in patients with liver cirrhosis, 20% decrease in cardiac index may not be meaningful in patients with cirrhosis and hyperdynamic hemodynamics with already elevated baseline cardiac output. Also, a previous study reported that there was a poor correlation between cardiac index and SvO_2_ during liver transplantation [36]. 

Several urinary or serum biomarkers were reported to be sensitive to predict AKI after surgery [37,38,39]. The performance of neutrophil gelatinase-associated lipocalin (NGAL) to predict AKI after surgery in terms of AUC was reported to be 0.82 to 0.83 after cardiac surgery [40]. The AUC of urinary NGAL on postoperative day one to predict AKI after liver transplantation was 0.79 [41]. Considering these AUC, the performance of our multivariable prediction could be regarded as similar to that of NGAL. In addition to NGAL, many other promising biomarkers have been reported including kidney injury molecule-1 (KIM-1), interleukin-18, liver-type fatty-acid binding protein (L-FABP), angiotensinogen [42], tissue inhibitor of metalloproteinase-2 (TIMP-2), and insulin-like growth factor-binding protein-3 (IGFBP-3) [38,43]. Two recent studies reported that biomarker-guided implementation of KDIGO interventions could decrease the incidence of AKI after cardiac and major abdominal surgery [44,45]. However, the performance of these biomarkers are still controversial [46] and there seems to be a long way to go before incorporating the biomarkers into our routine perioperative practice. 

Hemodynamic optimization may protect renal function in surgical patient [47]. Our results may suggest that hemodynamic goals should not be managed step-by-step—i.e., firstly, optimize preload; secondly, optimize contractility; thirdly, optimize afterload, and so on—and should be interpreted comprehensively. Goal-directed therapy algorithm usually establishes several hemodynamic goals first and do not pay attention to the remaining goals until the first goal, usually preload index, is achieved. However, hemodynamic goals including preload, afterload, and oxygenation index, as well as urine output, are associated with each other. Therefore, we suggest that intraoperative urine flow rate should be interpreted in conjunction with other hemodynamic parameters such as cardiac index or SvO_2_. However, routine use of pulmonary artery catheter has been a subject of debate. A previous multicenter trial reported no difference in mortality in high-risk surgical patients with and without hemodynamic management guided by pulmonary artery catheter [48]. Pulmonary artery catheter was associated with higher risk of pulmonary embolism. The use of less invasive monitoring with central venous oxygen saturation may substitute the SvO_2_ monitoring with pulmonary artery catheter [49]. 

There was a significant difference in gender distribution between the patients with and without AKI (Table 1). The incidence of female was significantly higher in the AKI group compared to the no-AKI group, which was consistent with a previous study [50], albeit not in other studies [1,20]. However, female was not an independent predictor in our multivariable analysis, suggesting that female gender is associated with other significant covariates. We compared the independent predictors identified in our multivariable analysis between male and female groups (Supplemental Table S3) and found that female was associated with significantly lower preoperative hemoglobin, and larger intraoperative crystalloid administration compared to male group in our study population. 

The present study had several limitations. Firstly, it was a single-center retrospective analysis, and urine flow rate data collected from the medical records may have been inaccurate. Furthermore, our cutoff for urine output may not be extrapolated to other institutions with different fluid management strategy and different baseline medical conditions, although multivariable adjustment was performed in this study. The intraoperative urine output may differ markedly depending on the intraoperative goal of fluid management, transfusion amount and intraoperative diuretics or hydroxyethyl starch use. Secondly, in our analysis, we used a mean urine flow rate during surgery rather than hourly urine output. However, oliguria lasting for longer than 6 h is required for the KDIGO criteria. Furthermore, there may be critical periods during LDLT, such as the anhepatic or reperfusion period, which involve unstable hemodynamics and severe metabolic acidosis [34]. In future studies, duration of oliguria as well as phases of LDLT when oliguria developed should be considered when investigating the association of oliguria with AKI. Different oliguria cutoffs should be considered including 1.0 mL/kg/h. Thirdly, we did not consider the duration of a decrease in hemodynamic variables. We evaluated the association between the decrease in hemodynamic variables for at least one measurement and AKI. Our electronic record has data of most hemodynamic variables in 5-min intervals. Further studies may investigate the possible dose-response relationship between the duration of hemodynamic deterioration and the risk of AKI. Fourthly, since our study was a retrospective analysis and intraoperative mean urine output was used for analysis, the temporal relationship between oliguria and decrease in hemodynamic variables could not be identified. The risk of AKI may be different between oliguria following deterioration in hemodynamic variables and oliguria with no temporal relationship hemodynamic derangement. 

## 5. Conclusions

Intraoperative oliguria alone could not accurately predict AKI after LDLT determined by creatinine criteria, although there were significant associations using a cutoff of oliguria <1.0, <0.5 and <0.3 mL/kg/h. However, when oliguria with these cutoffs was found with decreased intraoperative SvO_2_, the performance to predict AKI improved significantly and the predictive ability of multivariable prediction model was significantly enhanced. Decrease in cardiac index or mean arterial blood pressure combined with oliguria did not significantly increase the AUC to predict AKI after LDLT. Intraoperative oliguria interpreted in conjunction with a decrease in SvO_2_ may suggest the risk of AKI after LDLT more reliably in patients with normal baseline renal function and who did not receive hydroxyethyl starch during transplantation surgery.

## Figures and Tables

**Table 1:** Patient characteristics and perioperative parameters.

Characteristic	No AKI Group (*n* = 378)	AKI Group (*n* = 205)	*p*-Values
**Demographic data**			
Age, years	53 (47–58)	53 (49–58)	0.405
Female, n	85 (22.5)	63 (30.7)	0.029
Body-mass index, kg/m^2^	22.9 (21.0–24.8)	23.3 (21.6–26.1)	0.008
**Background medical status**			
Hypertension, *n*	39 (10.3)	12 (5.9)	0.069
Diabetes mellitus, *n*	47 (12.4)	22 (10.7)	0.544
Alcoholic liver cirrhosis, *n*	44 (11.6)	25 (12.2)	0.843
HBV hepatitis, *n*	159 (42.1)	71 (34.6)	0.080
HCV hepatitis, *n*	25 (6.6)	12 (5.9)	0.719
Hepatocellular carcinoma, *n*	209 (55.3)	113 (55.1)	0.969
Cholestatic disease, *n*	7 (1.9)	6 (2.9)	0.401
Preoperative hemoglobin, g/dL	11.5 (9.7–13.2)	10.2 (8.9–11.9)	<0.001
Preoperative serum albumin level, g/dL	3.0 (2.5–3.6)	2.8 (2.5–3.2)	0.002
Preoperative serum creatinine, mg/dL	0.90 (0.75–1.10)	0.85 (0.68–1.04)	0.014
Preoperative estimated glomerular filtration rate, mL/min	88 (70–109)	94 (75–122)	0.021
MELD score	13 (9–21)	17 (11–21)	0.001
Child-Turcotte-Pugh score	8 (6–11)	9 (7–11)	<0.001
Child class, n (A/ B/ C)	109 (33.5)/96 (29.5)/120 (36.9)	28 (16.2)/61 (35.3)/84 (48.6)	<0.001
Preoperative LVEF. %	65 (61–68)	65 (62–68)	0.254
Preoperative beta-blocker, *n*	29 (7.7)	13 (6.3)	0.645
Preoperative diuretics, *n*	16 (4.2)	12 (5.9)	0.382
**Donor/ graft factors**			
Age, years	31 (23–41)	30 (23–38)	0.302
Estimated GRWR	1.17 (1.01–1.46)	1.16 (1.00–1.41	0.396
ABO incompatible, *n*	19 (5.0)	10 (4.9)	0.937
**Operation and anesthesia details**			
Operation time, h	7.0 (6.1–8.2)	7.6 (6.5–8.6)	0.001
Cold ischemic time, min	71 (58–83)	78 (65–95)	<0.001
Warm ischemic time, min	31 (23–40)	32 (26–41)	0.142
Intraoperative furosemide use, *n*	71 (18.8)	52 (25.4)	0.063
Intraoperative furosemide dose, mg	0 (0–0)	0 (0–5)	0.040
Intraoperative use of epinephrine, *n*	148 (39.2)	81 (39.5)	0.933
Intraoperative dose of epinephrine, mcg	0 (0–10)	0 (0–5)	0.603
Intraoperative mean blood glucose, mg/dL	163 (144–178)	164 (145–183)	0.216
Intraoperative highest blood glucose, mg/dL	211 (194–230)	218 (200–238)	0.012
Mean trough level of tacrolimus during posmiddleerative first week (ng/mL)	6.2 (3.2–8.5)	6.5 (3.4–9.1)	0.098
**Bleeding and transfusion amount**			
pRBC transfusion, units	4 (0–10)	6 (3–12)	<0.001
FFP transfusion, units	4 (0–8)	6 (2–12)	<0.001
Blood loss per body weight, mL/kg	33 (18–64)	47 (23–91)	<0.001
**Input and output during surgery**			
Intraoperative average urine flow rate, mL/kg/h	1.36 (0.89–2.08)	1.15 (0.76–1.72)	0.007
Crystalloid administration, mL/kg	53 (37–73)	57 (40–85)	0.007
Net fluid balance during surgery, mL/kg	31 (13–52)	36 (18–59)	0.072

The values are expressed as the mean (standard deviation) or median [interquartile range] or number (%). AKI = acute kidney injury; MELD score = model for end-stage liver disease score; GRWR = graft to recipient body weight ratio; pRBC = packed red blood cell; FFP = fresh frozen plasma. Net fluid balance was calculated by total input subtracted by total output.

**Table 2:** Comparisons of performance of risk factors between the oliguria with different cutoffs alone, 20% decreased hemodynamic variables alone, and their combined variables.

Oliguria	Coupled Hemodynamic Variable	AUC (95% CI)	*p*-Value	Sensitivity	Specificity	PPV	NPV
<0.3	None	0.55 (0.50–0.60)	0.031	16.1	94.7	62.3	60.5
<0.5	None	0.60 (0.56–0.64)	<0.001	49.8	56.6	50.1	64.7
<1.0	None	0.55 (0.50–0.60)	0.040	53.7	48.7	38.3	69.8
No	SvO_2_	0.66 (0.61–0.70)	<0.001	57.1	79.1	59.7	77.3
No	Cardiac index	0.56 (0.52–0.61)	0.011	62.0	50.8	40.6	71.1
No	Mean arterial pressure	0.53 (0.48–0.58)	0.219	52.2	54.0	38.1	67.5
<0.3	SvO_2_	0.61 (0.56–0.66)	<0.001	11.3	97.4	82.5	50.0
<0.3	Cardiac index	0.55 (0.50–0.60)	0.046	16.1	93.9	58.9	67.4
<0.3	Mean arterial pressure	0.53 (0.48–0.58)	0.191	13.2	91.4	51.9	66.5
<0.5	SvO_2_	0.72 (0.68–0.75)	<0.001	59.1	86.5	69.6	78.8
<0.5	Cardiac index	0.56 (0.52–0.60)	0.011	37.6	75.1	45.0	68.9
<0.5	Mean arterial pressure	0.53 (0.49–0.57)	0.276	29.3	73.2	40.0	65.5
<1.0	SvO_2_	0.58 (0.53–0.63)	0.001	37.1	79.1	49.0	69.9
<1.0	Cardiac index	0.52 (0.47–0.57)	0.418	39.5	64.6	37.7	66.3
<1.0	Mean arterial pressure	0.50 (0.45–0.55)	0.929	30.2	69.3	34.8	64.7

AUC = area under the receiver operating characteristic curve, CI = confidence interval, SvO_2_ = mixed venous oxygen saturation, PPV = positive predictive value, NPV = negative predictive value. Coupled hemodynamic variables mean 20% decrease of the hemodynamic variables compared to baseline. AUC of the univariable analysis was reported. *P*-value are the results of testing the null hypothesis of AUC is 0.50.

**Table 3:** Multivariable logistic regression analysis to predict acute kidney injury after liver transplantation with and without oliguria or hemodynamic variables.

Variable	Odds Ratio	95% CI	*p*-Value
**Without urine output or hemodynamic variables**			
Body-mass index, kg/m^2^	1.09	1.03–1.16	0.004
Preoperative hemoglobin, g/dL	0.86	0.77–0.95	0.004
Preoperative albumin, g/dL	0.88	0.68–1.15	0.158
Operation time, h	1.07	0.94–1.22	0.123
Cold ischemic time, min	1.02	1.01–1.03	<0.001
Red blood cell transfusion, unit	1.10	1.05–1.12	0.010
Crystalloid administration, per 10 mL/kg	1.07	1.00–1.15	0.068
**Including urine output coupled with hemodynamic variables**			
Body-mass index, kg/m^2^	1.12	1.05–1.20	0.001
Preoperative hemoglobin, g/dL	0.89	0.79–0.99	0.041
Preoperative diuretics	1.68	0.58–4.89	0.139
Operation time, h	1.12	0.91–1.34	0.236
Cold ischemic time, min	1.02	1.01–1.03	0.003
Crystalloid administration, per 10 mL/kg	1.08	1.02–1.15	0.011
SvO_2_ reduction with oliguria <0.5 mL/kg/h	7.64	4.82–12.11	<0.001

Two separate logistic regression analysis was performed with and without intraoperative mean urine flow rate and intraoperative hemodynamic variables. CI = confidence interval. Stepwise backward variable selection was used and cutoff of *p* < 0.20 was used to select the variables.

## Supplementary Materials

The following are available online at http://www.mdpi.com/2077-0383/8/1/29/s1, Supplemental Table S1. Multivariable logistic regression analysis to predict acute kidney injury after liver transplantation without stepwise variable selection. Supplemental Table S2. Odds ratios (95% confidence intervals) and their P-values according to the categorized intraoperative urine flow rate with different cutoffs determined by both the univariable and multivariable logistic regression analysis for acute kidney injury. Supplemental Table S3. Subgroup analysis for the patients who had not received diuretics either before or during surgery (*n* = 435). Odds ratios (95% confidence intervals) and their p-values according to the categorized intraoperative urine flow rate with different cutoffs determined by both the univariable and multivariable logistic regression analysis for acute kidney injury were shown. Supplemental Table S4. Comparisons of independent predictors of the multivariable logistic regression analysis of our study between male and female. Supplemental Figure S1. Distribution of mean urine flow rate during liver transplantation surgery in all patients (upper), and box and whisker plots of urine flow rate during liver transplantation surgery with and without postoperative acute kidney injury (lower).
