# Supplementary material for: Intraoperative Oliguria with Decreased SvO2 Predicts Acute Kidney Injury after Living Donor Liver Transplantation
Source: J Clin Med. 2018 Dec 28;8(1):29. doi: 10.3390/jcm8010029 (PMC6351957; doi:10.3390/jcm8010029)

Supplemental Materials

Intraoperative oliguria with decreased SvO_2 predicts acute kidney injury after living donor liver transplantation

Supplemental Table S1. Multivariable logistic regression analysis to predict acute kidney injury after liver transplantation without stepwise variable selection.

Supplemental Table S2. Odds ratios (95% confidence intervals) and their P-values according to the categorized intraoperative urine flow rate with different cut-offs determined by both the univariable and multivariable logistic regression analysis for acute kidney injury of any stage in all patients.

Supplemental Table S3. Subgroup analysis for the patients who had not received diuretics either before or during surgery (n=435). Odds ratios (95% confidence intervals) and their p-values according to the categorized intraoperative urine flow rate with different cut-offs determined by both the univariable and multivariable logistic regression analysis for acute kidney injury were shown.

Supplemental Table S4. Comparisons of independent predictors of the multivariable logistic regression analysis of our study between male and female.

Supplemental Figure S1. Distribution of mean urine flow rate during liver transplantation surgery in all patients (upper), and box and whisker plots of urine flow rate during liver transplantation surgery with and without postoperative acute kidney injury (lower).

Supplemental Table S1. Multivariable logistic regression analysis to predict acute kidney injury after liver transplantation without stepwise variable selection.

Variable	Odds Ratio	95% CI	p-value
Age, year	1.01	0.98 – 1.05	0.441
Female	1.29	0.62 – 2.68	0.505
Body-mass index, kg/m^2	1.04	0.98 – 1.18	0.069
Year of operation, per 1 year increase	0.96	0.81 – 1.23	0.365
MELD score	1.08	0.93 – 1.24	0.478
CTP score	1.11	0.92 – 1.35	0.281
Hypertension	0.82	0.50 – 1.37	0.183
Diabetes Mellitus	1.47	0.59 – 3.66	0.216
Preoperative hemoglobin, g/dL	0.94	0.83 – 1.05	0.057
Preoperative albumin, g/dL	0.92	0.60 – 1.41	0.706
Preoperative serum creatinine, mg/dL	1.23	0.72 – 2.27	0.260
ABO type incompatibility	2.35	0.58 – 9.60	0.233
Operation time, hour	1.14	0.88 – 1.46	0.065
Cold ischemic time, min	1.03	1.00 – 1.09	0.052
Warm ischemic time, min	1.01	0.98 – 1.03	0.716
Intraoperative diuretics use	0.95	0.50 – 1.84	0.886
Intraoperative blood loss, per 100 ml/kg	1.24	0.10 – 15.77	0.870
Crystalloid administration, per 10 ml/kg	1.05	0.96 – 1.21	0.089
Colloid administration, per 10 ml/kg	1.39	0.99 – 1.04	0.207
Red blood cell transfusion, unit	1.16	0.95 – 1.36	0.962
Fresh Frozen plasma, unit	1.04	0.93 – 1.16	0.534
Intraoperative furosemide use	1.10	0.59 – 2.06	0.759
SvO_2 reduction with oliguria < 0.5 ml/kg/hr	3.22	1.44 – 6.98	0.001

CI = confidence interval; MELD score = Model for End-stage Liver Disease score; CTP score = Child-Turcotte-Pugh score; SvO_2 = Mixed venouos oxygen saturation.

Supplemental Table S2. Odds ratios (95% confidence intervals) and their P-values according to the categorized intraoperative urine flow rate with different cut-offs determined by both the univariable and multivariable logistic regression analysis for acute kidney injury of any stage in all patients.

Cut-off (mL/kg/h)	Unadjusted OR (95% CI)	*P*-value	Adjusted OR (95% CI)	*P*-value
<3.0	1.33 (0.71 – 2.50)	0.369	1.69 (0.65 – 4.35)	0.280
<2.5	1.33 (0.81 – 2.17)	0.265	1.48 (0.72 – 3.02)	0.288
<2.0	1.66 (1.09 – 2.53)	0.019	1.89 (0.82 – 3.51)	0.155
<1.5	1.59 (1.12 – 2.26)	0.009	1.61 (0.95 – 2.71)	0.075
<1.0	1.51 (1.07 – 2.13)	0.018	1.64 (1.00 – 2.74)	0.049
<0.5	2.69 (1.88 – 3.85)	<0.001	3.41 (1.93 – 6.02)	<0.001
<0.3	2.43 (1.61 – 3.16)	<0.001	2.33 (1.08 – 5.08)	0.002

OR = odds ratio, CI = confidence interval. In multivariable logistic regression analysis, all covariates in Table 1 were considered.

Supplemental Table S3. Subgroup analysis for the patients who had not received diuretics either before or during surgery (n=435). Odds ratios (95% confidence intervals) and their p-values according to the categorized intraoperative urine flow rate with different cut-offs determined by both the univariable and multivariable logistic regression analysis for acute kidney injury were shown.

Cut-offs (mL/kg/h)	Unadjusted OR (95% CI)	*P*-value	Adjusted OR (95% CI)	*P*-value
<3.0	1.12 (0.54 – 2.35)	0.757	1.92 (0.52 – 7.04)	0.326
<2.5	1.37 (0.74 – 2.53)	0.313	2.31 (0.85 – 6.27)	0.100
<2.0	1.71 (1.03 – 2.86)	0.039	2.66 (0.82 – 6.31)	0.126
<1.5	1.40 (0.93 – 2.10)	0.109	1.45 (0.76 – 2.77)	0.266
<1.0	1.11 (0.75 – 1.66)	0.597	1.18 (0.63 – 2.21)	0.612
<0.5	2.00 (1.31 – 3.05)	0.001	2.10 (1.05 – 4.20)	0.035
<0.3	2.34 (1.22 – 4.48)	0.010	2.54 (0.79 – 8.17)	0.119

OR = odds ratio, CI = confidence interval. In multivariable logistic regression analysis, all covariates in Table 1 were considered.

Supplemental Table S4. Comparisons of independent predictors of the multivariable logistic regression analysis of our study between male and female.

Variable	Male (n = 435)	Female (n = 148)	*P*-value
Body-mass index, kg/m^2	23.2 (21.5 – 25.2)	22.6 (20.8 – 25.4)	0.165
preoperative hemoglobin, g/dL	11.4 (9.5 – 13.2)	10.1 (9.0 – 11.5)	<0.001
preoperative albumin, g/dL	2.9 (2.5 – 3.6)	2.9 (2.5 – 3.3)	0.206
Operation time, hour	7.3 (6.2 – 8.3)	7.1 (6.1 – 8.2)	0.375
Cold ischemic time, min	72.1 (60.0 – 86.0)	72.2 (61.0 – 87.2)	0.807
RBC transfusion, unit	4 (1 – 10)	5 (2 – 11)	0.615
Crystalloid administration, per 10 ml/kg	5.3 (3.7 – 7.3)	5.9 (4.2 – 8.0)	0.024

RBC = red blood cell. *P*-values are the results of Mann-Whitney U test.

Supplemental Figure S1. Distribution of mean urine flow rate during liver transplantation surgery in all patients (upper), and box and whisker plots of urine flow rate during liver transplantation surgery with and without postoperative acute kidney injury (lower).

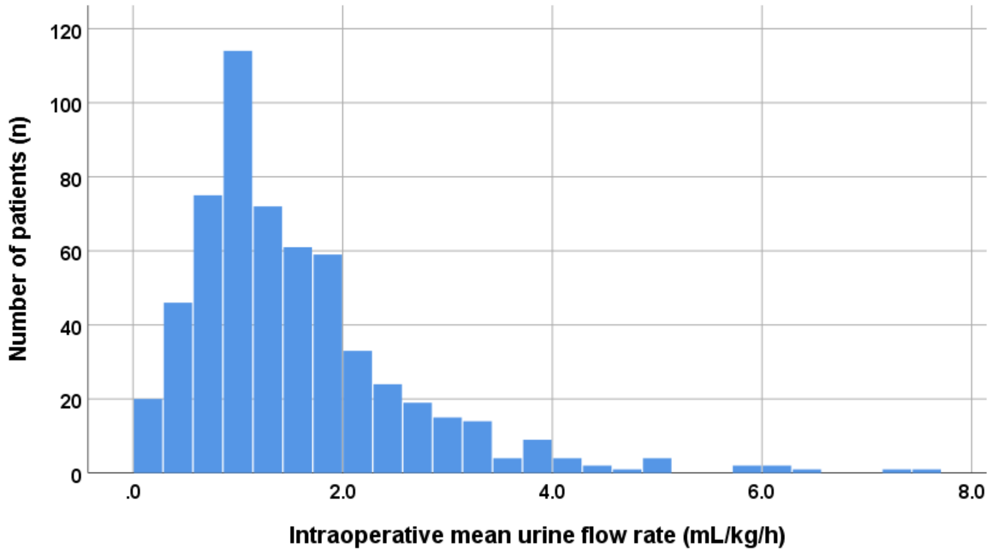

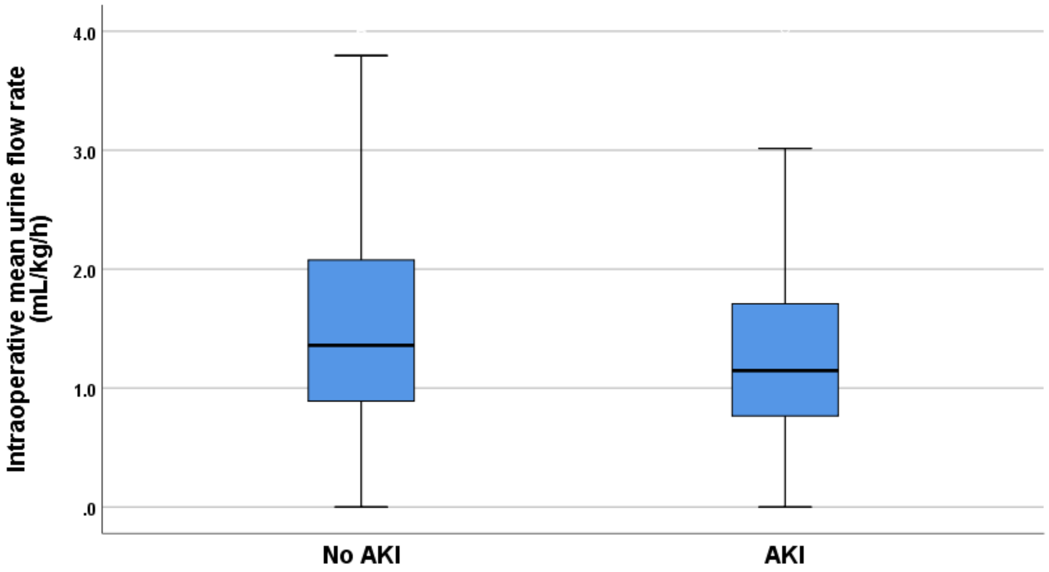

Supplement: Supplementary file 1 [file jcm-08-00029-s001.pdf]
